# Circulating Virus–Host Chimera DNAs in the Clinical Monitoring of Virus-Related Cancers

**DOI:** 10.3390/cancers14102531

**Published:** 2022-05-20

**Authors:** Chiao-Ling Li, Shiou-Hwei Yeh, Pei-Jer Chen

**Affiliations:** 1 Graduate Institute of Microbiology, College of Medicine, National Taiwan University, Taipei 100, Taiwan; chiaolingli@ntu.edu.tw; 2 Center for Genomic Medicine, College of Medicine, National Taiwan University, Taipei 100, Taiwan; 3 Department of Clinical Laboratory Sciences and Medical Biotechnology, College of Medicine, National Taiwan University, Taipei 100, Taiwan; 4 Graduate Institute of Clinical Medicine, College of Medicine, National Taiwan University, Taipei 100, Taiwan; 5 Division of Gastroenterology, Department of Internal Medicine, National Taiwan University Hospital, Taipei 100, Taiwan

**Keywords:** virus DNA integration, circulating tumor DNA (ctDNA), liquid biopsy, virus–host chimera DNA (vh-DNA)

## Abstract

**Simple Summary:**

Cell-free tumor DNA (ctDNA), the DNA released into circulation from tumors, is a promising tumor marker with versatile applications. The associations of the amount, somatic mutation frequency, and epigenetic modifications of ctDNA with the tumor burden, tumor behavior, and prognosis have been widely investigated in different types of tumors. However, there are still some challenging issues to be resolved before ctDNA can complement or even replace current serum tumor markers. We propose employing exogenous viral DNA integration that produces unique virus–host chimera DNA (vh-DNA) at junction sites. Cell-free vh-DNA may become a new biomarker because it overcomes background interference detection problems, takes advantage of virus tropism to localize the tumor, and acts as a universal marker for monitoring clonal expansion or tumor loads in tumors related to oncogenic viruses.

**Abstract:**

The idea of using tumor-specific cell-free DNA (ctDNA) as a tumor biomarker has been widely tested and validated in various types of human cancers and different clinical settings. ctDNA can reflect the presence or size of tumors in a real-time manner and can enable longitudinal monitoring with minimal invasiveness, allowing it to be applied in treatment response assessment and recurrence monitoring for cancer therapies. However, tumor detection by ctDNA remains a great challenge due to the difficulty in enriching ctDNA from a large amount of homologous non-tumor cell-free DNA (cfDNA). Only ctDNA with nonhuman sequences (or rearrangements) can be selected from the background of cfDNA from nontumor DNAs. This is possible for several virus-related cancers, such as hepatitis B virus (HBV)-related HCC or human papillomavirus (HPV)-related cervical or head and neck cancers, which frequently harbor randomly integrated viral DNA. The junction fragments of the integrations, namely virus–host chimera DNA (vh-DNA), can represent the signatures of individual tumors and are released into the blood. Such ctDNA can be enriched by capture with virus-specific probes and therefore exploited as a circulating biomarker to track virus-related cancers in clinical settings. Here, we review virus integrations in virus-related cancers to evaluate the feasibility of vh-DNA as a cell-free tumor marker and update studies on the development of detection and applications. vh-DNA may be a solution to the development of specific markers to manage virus-related cancers in the future.

## 1. Introduction

In 2018, infection caused approximately 13% of cancer cases worldwide [1]. The two well-known oncogenic viruses, hepatitis B virus (HBV) and high-risk human papillomavirus (HPV), account for 47% of infection-induced tumors [2]. HPV and HBV promote carcinogenesis through the production of oncogenic viral proteins, modulation of the immune microenvironment, or disposition of insertional mutagenesis in the host genome by viral DNA integration. In this review, we will focus on viral integration events relevant to carcinogenesis and introduce the idea of exploiting viral DNA traces in the human genome as a cell-free tumor marker.

## 2. Cancers and Viral DNA Integration

### 2.1. Hepatitis B Virus-Related Hepatocellular Carcinoma (HBV-Related HCC)

HBV infection in adults can be cleared mostly as an acute infection; however, infection in neonates through vertical transmission can easily become a chronic infection. HBV enters hepatocytes using sodium taurocholate cotransporting polypeptide (NTCP) as the receptor, releases its nucleocapsid in the cytoplasm, and has relaxed circular DNA repaired into covalently closed circular DNA (cccDNA) by cellular mechanisms in the nucleus. cccDNA is the template for viral DNA replication and an HBV reservoir after active replication is stalled by antiviral treatment. HBV DNA integrates into the chromosomes of infected hepatocytes in the form of double-stranded linear DNA (dslDNA), a by-product created from in situ priming during positive-strand DNA synthesis. DslDNA integrates into the hepatocyte genome probably through non-homologous end-joining or microhomology-mediated end-joining DNA-repair mechanisms.

HBV DNA integrates into random sites throughout human genome during infection [3]. The insertion of HBV DNA may result in the production of viral proteins, generation of virus–human transcripts such as the hybrid RNA transcript of the human LINE1 and the HBV-encoded X gene (HBx-LINE1), or activation of adjacent host gene transcription by virus enhancers/promoters such as the reactivation of *TERT*. The excessively high clonal integration rate (~90%) observed in HBV-related HCCs supports integration provides a selective advantage for virus-infected hepatocytes in carcinogenesis.

Integrated HBV DNA may enhance carcinogenesis via three mechanisms. First is the overexpression of oncogenic viral protein. HBx is frequently C′-terminal truncated and can be observed in 80% of HBV-related HCCs [4], which are associated with increased invasiveness and metastasis. HBx is known to promote HBV transcription by inducing the degradation of the smc5/6 complex through DDB1-containing E3 ubiquitin ligase [5], which contributes to chronic HBV infection. In addition to the persistence of the inflammatory environment, HBx also contributes to carcinogenesis by inhibiting DNA homologous recombination repair [6], anti-apoptosis, and epigenetic regulation.

The second mechanism relates to the repetitive sequences in the human genome, long interspersed nuclear element (LINE1) retrotransposon. Integrated HBV DNA was shown to generate a novel HBx-LINE1 transcript in 23% of HBV-related HCCs. HBx-LINE1 promotes epithelial–mesenchymal transition (EMT) through activation of Wnt/β-catenin signaling, and is associated with poor survival [7]. It can also promote mitosis and liver injury by sequestration of miR-122 [8].

The third mechanism is insertional mutagenesis by HBV DNA integration. Whole-genome sequencing (WGS) of HBV-related HCCs identified *TERT*, *MLL4* (*KMT2B*), and *CCNE1* as the most reproduced hotspot genes with HBV integrations. HBV-*TERT*, HBV-*MLL4*, and HBV-*CCNE1* can be found in approximately 25%, 10%, and 3% of HBV-related HCC cases, respectively. The selection of hotspot integrations in tumors indicates that these genes contribute to carcinogenesis. HBV integration in the promoter region of *TERT* results in significant upregulation of transcription from a tightly and sophisticatedly regulated low level in normal hepatocytes. As the limiting factor for telomerase complex activity, the expression of TERT enables telomere length maintenance, thus preventing the cell from replicative senescence. TERT can also contribute to carcinogenesis in enzymatic activity-independent manners, such as promoting β-catenin transcriptional activity through BRG1 [9], amplifying NF-κB-dependent transcription [10], or inhibiting P53 or BCL-2 [11,12]. Rather than being a promoter, HBV integrates into introns 3-5 in *MLL4*, an H3K4 histone methyltransferase gene, leading to overexpression of the N′-terminal truncated MLL4 protein [13]. Although the function of N’-terminal truncated MLL4 in carcinogenesis has not yet been fully elucidated, it is clear that H3K4 methylation increases in HBV-related HCCs with HBV-*MLL4*, which in turn can increase overexpression of oncogenic genes. The level of cyclin E expression was also drastically increased by the integration of HBV in *CCNE1*, and the HCC positive for *CCNE1* integration was characterized by high proliferation activity and inactivation of RB1 and PTEN [14]. Additionally, 40–50% of HBV integration is associated with copy-number variation or gene rearrangement, which can also promote carcinogenesis through alteration of proximal and distal tumor-driving genes [15].

When we examine integration at the nucleotide level, each integration is unique due to the combination of virus and host genome, including the differences in the position of breakpoint, the in–del or local rearrangement around breakpoint, and the sequence variation in both HBV and the human genome. Therefore, the HBV integration sequence can be used as a highly specific genetic marker for individual hepatocytes. Given the carcinogenesis process, the fact that integration occurs during virus infection places this unique genetic change as an early event. HBV integration is likely the trunk mutation or driver mutation for carcinogenesis, and can act as a permanent genetic marker that remains in the hepatocyte undergoing clonal expansion and subsequent tumor growth.

### 2.2. Human Papillomavirus (HPV)-Related Tumors

HPV infects basal cells of the epithelium through intimate contact in humans. There are more than 200 types of HPV, most of which cause benign diseases such as warts, but so-called “high-risk” HPVs are known to be the cause of ~90% of cervical cancer cases worldwide [16]. HPV-16 and HPV-18 account for approximately 70% of cancers in the cervix, vagina, and anus, and for approximately 30–40% of cancers of the vulva, penis, and oropharynx [17].

As in HBV, HPV DNA integration is not required to complete the viral life cycle, and integrated HPV DNA cannot produce progeny. The association between HPV DNA integration and carcinogenesis was shown by the increase in integration-positive cells during the histological progression from cervical intraepithelial neoplasia (CIN) I to III [18,19]. At the tumor stage, integration was detected in 80–100% of HPV-positive cervical cancers [20,21,22], 38–80% of anal carcinomas [23,24,25], and 30–70% of HPV-positive head and neck cancers [26,27,28,29].

The integration of HPV follows two patterns, one with direct integration of an incomplete single viral genome and the other with looping of multiple genomes [30]. The integration usually causes the disruption of viral E1 and E2 genes and thus alleviates the inhibited transcription of carcinogenic E6 and E7 genes [31,32]. Additionally, the virus–host transcript is more stable than the viral coding E6 or E7 transcript [33]. The resulting increased expression of E6 and E7 can promote the degradation of P53 and RB to inhibit apoptosis [34]. However, in contrast to HBV-related HCCs, no common HPV integration hot spots have been identified [35,36,37,38]. Therefore, the carcinogenic role of HPV integration depends more on the production of oncogenic viral protein rather than the intervention in the expression of the integration flanking genes.

### 2.3. Other Human Cancers with Viral Integrations

Integration has also been observed in other oncogenic DNA viruses, such as Epstein–Barr virus (EBV), Kaposi’s sarcoma-associated herpesvirus (KHSV/HHV-8), and Merkel cell polyomavirus (MCPyV). Nevertheless, studies of the oncogenic mechanism of these viruses generally focus on oncogenic viral proteins, virally encoded transcripts, and cofactors, such as the prerequisite for somatic mutations or coinfection with other pathogens. The detailed characteristics of integration of these viruses in clinical samples remain to be investigated. For example, although EBV usually persists in the episomal form in tumors, integrated EBV DNA is detectable in 8–36% of EBV-positive tumors revealed by Southern blotting and fluorescence in situ hybridization [39]. An analysis of the RNA sequence across 15 EBV-related malignancies showed that EBV DNA integration is enriched in open chromatin of the host genome, and may upregulate the expression of integration-flanking genes [40]. However, the prevalence of integration in each type of tumor, the pattern of integrated viral DNA, the hotspot integration sites in the host genome, and the possible role of viral DNA integration in carcinogenesis, remain to be consolidated with a larger sample size in the era of next-generation sequencing (NGS). As more tumor samples were sequenced in HCC, it was discovered that adeno-associated virus (AAV) also integrates into the *CCNA2*, *CCNE1*, and *TERT* genes in human HCCs, suggesting that in rare circumstances, AAV integration may cause insertional mutagenesis [41].

Human T-lymphotropic virus type 1 retrovirus (HTLV-1) is also a well-recognized oncogenic RNA virus. HTLV-1 tends to integrate into transcriptionally active regions in human genome [42]. In asymptomatic HTLV-1 carriers, HTLV-1-infected T-cell clone is polyclonal. The change in clonality into oligoclonal/monoclonal is recognized as a risk factor for the onset and progression of adult T-cell leukemia–lymphoma (ATL) [43,44]. However, the selection of HTLV-1-infected T-cell clones in vivo is poorly understood, and has been proposed to be dependent on the radial intranuclear position of the integration, its genomic distance from the centromere, and the intensity of local host genome transcription [45]. Yet, the importance of integrated host gene is rather limited in carcinogenesis because no common integration hotspot was observed in T-cell clones in ATL patients [46]. Nevertheless, the integrated HTLV-1 sequence has been applied as a marker in assessing clonal size, progression from myelopathy/tropical spastic paraparesis (HAM/TSP) to ATL, and response to treatment [47,48,49].

## 3. From Circulating Cell-Free DNA to Cell-Free Tumor DNAs

### 3.1. Properties of Cell-Free DNA (cfDNA)

Discovered in 1948, circulating cell-free DNA (cfDNA) is defined as DNA fragments in circulation without association with cells [50]. cfDNA is detectable in blood, stool, urine, saliva, cerebrospinal fluid, ascetic fluid, and amniotic fluid samples [51]. Released from the cells through mechanisms that include apoptosis, necrosis, and extracellular vesicle secretion, cfDNA is fragmented DNA with a general size of 160–200 bp. Since cfDNA is fragmented, its half-life is short, ranging from 16 min to a few hours [52]. The concentration of cfDNA in the blood of a healthy subject is 10–30 ng/mL, but it can increase in those with conditions such as pregnancy, tumor growth, and inflammatory disease [53,54]. In recent years, the most well-known application of cfDNA has been non-invasive prenatal testing (NIPT)—the detection of fetus trisomy in maternal blood. Fetal cfDNA can be released from the placenta and detected as early as 7 weeks, when the fetus size is approximately 1.3 cm [55,56,57]. In a prospective study involving 15,841 pregnancies, the detection of trisomy 21 by a cfDNA test further improved the AUC to 0.999 in comparison to the AUC of 0.958 for traditional detection methods [58]. An even larger-scale study that included 21,172 pregnancies found that the detection sensitivity and specificity were 99.64 and 99.96, respectively [59]. The results clearly demonstrated the great power of cfDNA in detecting copy-number variation.

### 3.2. Cell-Free Tumor DNA (ctDNA) as a Tumor Marker for Detection, Residual Disease, or Monitoring Treatment Responses

The cfDNA released from the tumor during its turnover is called cell-free tumor DNA (ctDNA). ctDNA is also called ‘liquid biopsy’ because it provides information on genetic changes in tumors, including somatic mutations, copy-number variation, gene fusion, and epigenetic regulation from blood or body fluid instead of a tumor biopsy. Due to its characteristic short half-life and the low invasiveness of sampling, ctDNA has become a promising tumor marker that can reflect the tumor burden in a real-time manner and is suitable for long-term follow-up.

The application of ctDNA in monitoring tumor progression or emerging resistance to chemotherapies has been widely investigated [60,61,62,63]. The study by Dawson et al. [60] detected somatic mutations in cfDNA from 30 breast cancer patients receiving systemic treatment. When comparing the quantitative results of the ctDNA with the level of the CA15-3 breast cancer tumor marker, the decrease in ctDNA copy number reflected the treatment response, and ctDNA showed a better association with tumor burden than CA15-3. The detection sensitivity was 97% for ctDNA, which is higher than the 78% of CA15-3 in this study. In a later study also in breast cancer conducted by Garcia-Mrillas et al. [61], the authors identified tumor-specific mutations from tumor gDNA via high-depth, targeted, massively parallel sequencing (MPS). Droplet digital PCR (ddPCR) was applied for the detection of the point mutation in the plasma collected at 2–4 weeks after tumor resection to detect minimal residual disease (MRD). They found that the presence of point mutations in a single postoperative cfDNA was associated with early recurrence. Further tracking of ctDNA in serial samples can even identify recurrence 8 months before clinical diagnosis [61]. These proof-of-concept studies revealed the great potential of using tumor-specific somatic mutations as a cell-free tumor marker in the development of precision medicine. The applications of ctDNA in virus-related cancers are listed in Table 1.

### 3.3. Limitations of Using Somatic Mutations in ctDNA as Tumor Markers and Possible Solution: Virus–Host Chimera DNA (vh-DNA)

Despite promising preliminary results, there are limitations of using tumor-specific point mutations as cell-free tumor markers. First, the dramatically high amount of wild-type background cfDNA released from normal tissue interferes with the detection of ctDNA. Although the level of ctDNA increases proportionally to tumor burden or tumor aggressiveness, it usually accounts for less than 1% of total cfDNA. Taking a 70 kg man with a 100 g tumor, if the tumor releases ctDNA 10× faster than normal tissue, then cfDNA may account for 1.4% of the total cfDNA. Reliable detection of ctDNA by point mutations requires techniques with high specificity and sensitivity. Absolute quantification will be more suitable than relative quantification, since detection results may need to be compared in serial samples. Therefore, the detection of tumor-specific mutations in cfDNA requires techniques such as ddPCR, which can detect 0.01% of mutations in the wild-type background and achieves absolute quantification at the same time. Nevertheless, even with ddPCR, the detection of somatic mutations may still be insufficient in preliminary studies [84]. The detection sensitivity appeared to have improved in a subsequent study, but the coherence between tumors and cfDNA has not been reported [85].

The second limitation is the coverage of mutations for a specific type of cancer. The dominant mutation in HCC is *TERT* promoter mutation, G(-124)A or G(-146)A from ATG, which can be identified in 30–60% of HCC. The mutation rate limits the use of *TERT* promoter mutations as a ctDNA marker for up to 60% of HCC patients. When the tumor-specific mutation is unknown, common tumor-related mutations will be targeted for tumor detection by NGS. Approaches such as increased output for higher read depth, targeted NGS, unique molecule identifiers (UMI) in library preparation, or circulating single-molecule amplification and resequencing technology (cSMART) will be needed to justify mutation-calling for the detection of ctDNA. Some groups have detected multiple point mutations using targeted NGS to maximize detection sensitivity and the spectrum of the mutations [63,86,87]. Labgaa et al. performed ultradeep targeted NGS with a read depth of 5500× for the exons of 58 genes from HCC [88]. Using this approach, they identified 21 somatic mutations in six of eight tumors, and 71% of the mutations were detected in the corresponding cfDNA. Lin et al. conducted ultradeep targeted NGS of the coding exons of 466 genes and introns of 36 genes for both multifocal HCC and blood collected before tumor resection [83]. The identified driver mutations in tumors were detected in 63% of the corresponding cfDNA, with a read depth of 5397×.

Third, the somatic mutation may not be tumor-type-specific, so the localization of the tumor becomes a problem when using prevalent point mutations in cfDNA for primary tumor detection. For example, *TERT* promoter mutations can be found not only in HCC, but also in central nervous system, thyroid, bladder, and skin cancers [89]. It is difficult to differentiate the type of tumor only by ctDNA somatic mutations, but this issue can be solved by the unique pattern of methylation of ctDNA from different types of tissues [90,91].

**Figure 1:**
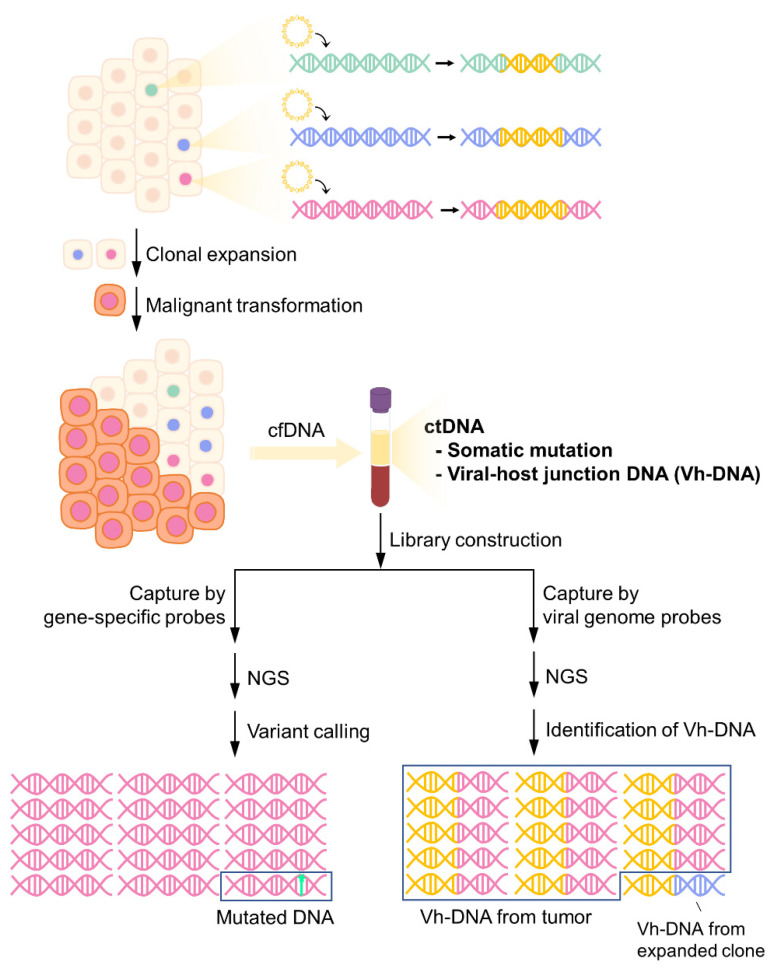
Detection of somatic mutations and vh-DNA from cfDNA by targeted-NGS. Virus DNA integrates into the host genome during infection. Infected cells may undergo clonal expansion due to insertional mutagenesis and eventually become tumor cells through the accumulation of somatic mutations. As cell-free tumor markers, both tumor-specific somatic mutations and vh-DNA can be detected in plasma. However, the frequency of somatic mutations is low in cfDNA, while tumor-released vh-DNA is the main component of the total population of vh-DNA.

To overcome the problems in using point mutations as cell-free tumor markers, we propose using junction fragments of viral DNA integration, namely virus–host chimera DNA (vh-DNA), as a cell-free tumor marker. During tumor turnover, the integrated viral DNA in the human genome is fragmented and released, allowing tumor-specific vh-DNA to be detected as ctDNA. There are several advantages to using vh-DNA as a cell-free tumor marker. First, the detection of vh-DNA is not impeded by a high level of wild-type background in the total cfDNA because the targeted sequence partly originates from exogenous viral DNA, which is easier to differentiate than a single point mutation (Figure 1). Second, the vh-chimera DNA can cover more patients than point mutations in virus-related tumors because the virus integration rate is much higher than the common point mutations. Third, the junction sequence created by virus integration and the flanking host gene sequence are different at the nucleotide level between tumors. Such differences allow vh-DNA to act as a unique barcode for individual tumors and may be applied in studies of tumor heterogeneity or determination of the clonal origin of recurrence. Fourth, when applied in the screening of unknown tumors in healthy subjects, the detection of vh-DNA indicates the location of the tumor according to virus tropism.

### 3.4. Proof-of-Concept Studies of Circulating vh-DNA in HBV-Related HCC

Given the advantages of vh-DNA, we conducted a proof-of-concept study to demonstrate the possibility of using vh-DNA as a tumor marker for HBV-related HCC [92]. We identified the HBV integration junction sequence from 44/50 (88%) HBV-related HCC samples using an in-house capture-NGS platform targeting HBV. With the acquired junction sequence at integration, we set up a tumor-specific vh-DNA ddPCR assay for each tumor and quantified the vh-DNA in the plasma samples before and after tumor resection. The targeted vh-DNA was detected by ddPCR in 43/44 (98%) of the plasma samples collected before resection, and the copy number of vh-DNA in plasma was associated with tumor burden, with an estimated detection limit of 1.5 cm. In comparison to the common serum tumor marker AFP, vh-DNA has a higher sensitivity in tumors <5 cm. The copy number of tumor-specific vh-DNA decreased in the plasma collected 2 months after resection, clearly reflecting the removal of the tumor by resection. Furthermore, the presence of tumor-specific vh-DNA in post-resection plasma is associated with early recurrence within one year. We also demonstrated that the integration could indicate whether the recurrence originated from the primary tumor clone or a de novo clone by direct capture-NGS of the plasma collected at the recurrence. In this study, we established the potential of vh-DNA as a circulating biomarker for HBV-related HCC.

Chen et al. performed HBV-targeted NGS to detect HBV integration in tumor and nontumor paired tissue and cfDNA [93]. The 29 integrations detected in plasma cfDNA can all be detected in tumor tissue, and its level in cfDNA correlated to the abundance of specific integrations in the tumor. They further validated the chimeric RNA transcript in four HCC, supporting that the HBV integration detected in cfDNA originated from the tumor. This study also indicated that a sufficient amount of probe is needed to detect the integration of HBV in cfDNA.

Using another source of cfDNA, Lin et al. showed that the HBV–host junction sequence (HBV-JS) can be identified in formalin-fixed paraffin-embedded (FFPE) tumor tissue and in urine [94]. This study verified the presence of tumor-specific HBV-JS in urine cfDNA from 5/8 (62.5%) patients using nested PCR and Sanger sequencing. In addition to cancer patients, they also detected HBV-JS in the urine of HBV-infected patients with hepatitis or cirrhosis using targeted NGS. However, the integrations identified in serial samples from the same patients were different, which may be due to their cutoff for detection. Nevertheless, this study showed that HBV-JS DNA is detectable in urine cfDNA in patients with hepatitis and cirrhosis.

Zheng et al. detected HBV integration breakpoints by targeted NGS in 481 HCC and 517 cirrhosis patients [95]. They also showed that the HBV integration breakpoints can be detected in cfDNA from patients with cirrhosis and HCC. Compared to cfDNA from HCC patients, both the number of integration breakpoints and the rate of hotspot integration, HBV-*TERT*, are significantly lower in cfDNA from patients with cirrhosis. They found that the integration breakpoints that can be detected in both tumor and corresponding cfDNA have a higher read frequency than the unmatched integration breakpoints, indicating that the matched breakpoints detected in cfDNA may result from clonal expansion in HCC. This result is consistent with a study that identified that the supporting reads for integration were higher in tumors than in hepatitis tissue [96].

### 3.5. Proof-of-Concept Studies of vh-DNA in HPV-Related Tumors

For HPV, there have been some case reports using integration junction sequences as proof of the tumor origin. Case studies have applied HPV integration and viral transcripts as markers to confirm brain metastases originating from HPV-positive oropharyngeal squamous cell carcinoma [97], ovarian metastases originating from endocervical adenocarcinoma [98], and tongue tumors derived from anal cancer [99]. Similarly, but aiming for an earlier pathological stage, a study by Hoyer et al. detected recurrence of CINs using HPV integration identified from swab samples taken before or at CIN3 surgery [100]. The viral–cellular junctions (vcj) were then detected in the follow-up swab by a qPCR assay. The results showed that the vcj is highly specific for recurrence, yet the sensitivity is rather limited, possibly due to the intratumoral heterogeneity of HPV integrations in CINs. This assumption may be verified if the researchers detect the clonality of recurrent CINs by NGS as in baseline swab samples. In their study, integrations were detected in only 10% of HPV-positive CINs but 80% of HPV-positive cervical cancers [101], supporting that the cervical cancers were derived from clonal expanded integration-positive CINs.

A few studies have tested the possibility of using HPV–human junction sequences as tumor cfDNA markers. An early study showed that the HPV–human junction sequence can be detected in the cfDNA of 85% of patients with stage II–IV tumors by qPCR, and its level in cfDNA was decreased by treatment and increased at recurrence [102]. Another study by Carow et al. aimed to investigate intratumor heterogeneity in different cervical cancer microdissections. They detected tumor-specific junction fragments in 23% of preoperative plasma samples using semi-nested PCR, and found that the presence of junction fragments in preoperative plasma was associated with lower recurrence-free survival [103]. The study by Sastre-Garau et al. performing CaptHPV examined the HPV integration in pairs of tumor and blood samples [104]. They identified 15 genotypes of HPV in HPV-related tumors, including cervical, anal, vulvar, oropharyngeal, and oral tumors. The same junction sequence was identified by CaptHPV in both tumors and ctDNA in 68% (34/54) of tumor–plasma pairs. The results showed that vh-DNA is also applicable as a cell-free tumor marker for HPV-related tumors. Another recent study by Jeannot et al. showed the detection rate of HPV integration by ddPCR is 39% (9/23), which is lower than the paralleled quantified HPV in 70% (16/23) of the cervical cancer patients [74].

**Figure 2:**
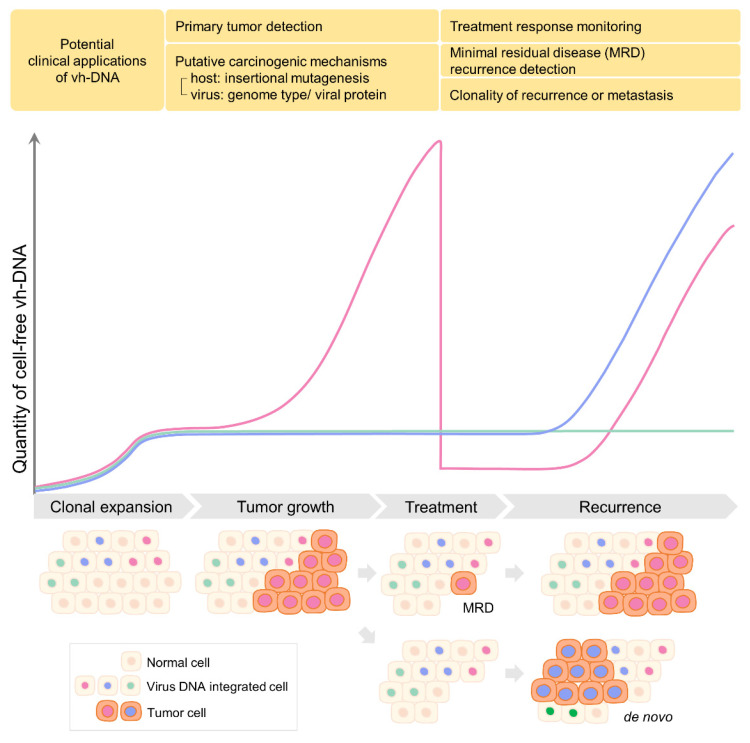
The potential applications of vh-DNA in tumor diagnosis and prognosis.

In summary, the approaches to detect vh-DNA in cfDNA are listed in Table 2. NGS-based approaches can detect vh-DNA without knowing the sequence of vh-DNA, but their sensitivity is limited. PCR-based assays are much more sensitive than NGS, yet detection may be interfered with due to the repetitive sequences in human genome, for example, human endogenous retrovirus sequence, which increases difficulties in designing specific primer sets. The potential applications of vh-DNA as a cell-free tumor marker during tumor progression and the course of treatment are illustrated in Figure 2.

## 4. Discussion

In basic research, the vh-DNA level can be applied as an indicator to evaluate the release of ctDNA into circulation in experimental models (Table 3). From the mechanistic view, when viral DNA integration occurs immediately upstream of the oncogenic genes, the insertional mutagenesis can activate downstream signaling pathways and thus promote the cell to undergo clonal expansion. Therefore, vh-DNA is also a marker for the activation of distinct insertional mutagenesis mechanism(s) in the expanding cells. The association between viral integration at specific gene(s) and the characteristics of the microenvironment and disease prognosis is worthy of investigation, for example in a proper immune-competent animal model [105]. The results may support the potential application of vh-DNA as a surrogate marker to predict the clinical outcome and even guide specific targeted treatment(s) for patients at early carcinogenic stages, before tumor development.

## 5. Conclusions

At the current stage, the feasibility of using vh-DNA as a clonal-specific, cell-free tumor marker for HBV and HPV-related tumors has been verified. Evaluation of therapeutic response, follow-up for MRD, determination of metastasis, and investigation into the association between cell-free vh-DNA and prognosis can be achieved by detecting vh-DNA in cfDNA. The side-by-side comparison between vh-DNA, somatic mutations, and the current routinely used serum tumor markers should be demonstrated in future studies to evaluate the sensitivity and specificity differences between the markers. It is also noteworthy that HBV integration can be detected in 5–10% of HCV-related HCC from HBV endemic areas, indicating resolved HBV infection in these patients. Vh-DNA resulting from HBV infection can be ctDNA for most HBV-HCC and certain HCV-related HCC patients in endemic areas.

The real challenge for vh-DNA is to detect sequence-unknown vh-DNA directly from cfDNA. This is required for the early detection of primary tumors and the emerging de novo tumors during follow-up after treatment. A highly sensitive NGS approach needs to be merged with a standardized workflow from sample collection, processing, library preparation, target enrichment, and the sequence analysis pipeline. Since vh-DNA is a marker for clonal expansion, it is also detectable in circulation at the stage before tumor development, possibly from the cirrhotic nodules undergoing clonal expansion. Therefore, vh-DNA will not be able to identify the stage of tumor or differentiate the viral-DNA integrated into normal or pre-cancerous tissues from the tumor tissues. Setting up the cutoff value to differentiate the tumor-specific vh-DNA from non-tumor-tissue-released vh-DNA is critical for achieving clinically significant sensitivity and specificity. A population study should be conducted that includes healthy subjects and patients in different stages of disease. Well-known tumor-related somatic mutations should also be incorporated to compensate the detection of tumors without viral DNA integration.

## Figures and Tables

**Table 1:** Applications of ctDNA in virus-related cancers.

Tumor	Application	Target	Approach	Patient	Results	Ref.
HCC	Tumor detection	Fragmentomic features	WGS	Training set: 159 HCC, 26 ICC, 7 cHCC-ICC, 170 control Test set: 157 HCC, 26 ICC, 6 cHCC-ICC, 164 control	Sensitivity 96.8% Specificity 98.8%	[64]
HCC	Tumor detection	5-hmc	5hmc-Seal profiling	Training set: 335 HCC, 263 CHB/LC, 522 control Validation set: 809 HCC, 129 CHB/LC, 256 control	Sensitivity 82.7% Specificity 76.4%	[65]
HCC	Tumor detection	5-hmc, nucleosome footprint, motif, and fragmentation profile	5-hmc sequencing, WGS	Training set: 255 HCC, 347 LC, 260 control Validation set: 95 HCC, 100 LC, 100 control Test set: 131 HCC, 1800 LC, 116 control	Sensitivity 95.4% Specificity 97.8%	[66]
HCC	Tumor detection	DNA methylation	Bisulfite sequencing	Training set: 120 HCC, 92 LC, 290 control Validation set: 7 HCC, 111 LC, 242 control	Sensitivity 84% Specificity 96%	[67]
CC	Prognosis prediction	PIK3CA mutation	ddPCR	117 CC	Presence of PIK3CA mutation in cfDNA associated with decreased DFS and OS.	[68]
HCC	Prognosis prediction	Somatic mutations	Targeted NGS	41 HCC	Median VAF of mutations in preoperative ctDNA was an independent predictor of RFS.	[69]
HNSCC	Prognosis prediction	Somatic mutations	Targeted NGS	75 HNSCC	Presence of somatic mutation in baseline ctDNA associated with decreased OS.	[70]
HNSCC	Prognosis prediction	Somatic mutations	ddPCR	18 HNSCC	Presence of somatic mutation in cfDNA after initial curative treatment associated with recurrence and decreased OS.	[71]
HNSCC	Prognosis prediction	Somatic mutations, DNA methylation	CAPP-seq, cfMeDIP-seq	30 HNSCC, 20 control	Baseline cfDNA with somatic mutation or HNSCC-specific methylation pattern associated with worse OS. Lack of post-treatment ctDNA clearance associated with recurrence.	[72]
CC and oropharynx cancer	Prognosis prediction	HPV	HPV-seq	33 CC, 13 oropharynx cancer	End-of-treatment timepoint cfDNA for recurrence prediction: sensitivity 100%, specificity 67%.	[73]
CC	Prognosis prediction	HPV	HPV E7 ddPCR	94 HPV-related CC	HPV ctDNA in the cfDNA at the end of treatment associated with a longer PFS.	[74]
HCC	Prognosis prediction	CNV and TFx quantification	WGS	64 HCC (TACE), 57 LC, 32 control	The change in TFx between pre-TACE and post-TACE cfDNA could predict patients’ PFS.	[75]
CC	Prognosis prediction Evaluation of treatment response	Somatic mutations	Targeted NGS	82 CC	PIK3CA, BRAF, GNA11, FBXW7, and CDH1 mutation in cfDNA associated with shorter PFS and OS. The decrease in mutations reflects treatment response.	[76]
CC	Evaluation of treatment response	Somatic mutations	Targeted NGS	24 CC	Change in mutation allele frequency in cfDNA can be observed during follow-up after treatment.	[77]
CC	Evaluation of treatment response	Somatic mutations	Targeted NGS	57 CC	The deviation in allele fraction in cfDNA reflects tumor volume.	[78]
HCC	Evaluation of treatment response	Somatic mutations	WGS	24 HCC (Lenvatinib)	The specificity and sensitivity of the reduction in the mean VAF in cfDNA to predict the partial response were 0.67 and 1.0	[79]
HCC	Evaluation of treatment response	TERTp mutation	ddPCR	67 HCC (32 TACE, 35 TKI)	The changes in hTERT promoter mutant DNA fraction in cfDNA indirectly reflect the amount of tumor necrosis during TACE and TKI therapy.	[80]
HNSCC	Detection of minimal residual disease	Somatic mutations	Targeted NGS	17 HNSCC	Tumor-specific somatic mutations can be detected in cfDNA before clinical recurrence.	[81]
HCC	Investigation of the intratumor heterogeneity in multinodular HCC	Somatic mutations	Targeted NGS	5 intrahepatic metastasis and 2 multicentric HCCs	CfDNA was able to capture not only clonal mutations but also the subclonal mutations detected in only one of the multiple biopsied nodules.	[82]
HCC	Investigation of the intratumor heterogeneity in multinodular HCC	Somatic mutations	Targeted NGS	11 multifocal HCC	Truncal mutations and the level of genomic heterogeneity could be identified by targeted NGS panel in patients with resected multifocal HCC.	[83]

Abbreviations: HCC, hepatocellular carcinoma; WGS, whole genome sequencing; ICC, intrahepatic cholangiocarcinoma; cHCC-ICC, combined hepatocellular and intrahepatic cholangiocarcinoma; 5-hmc, 5-hydroxymethylcytosine; CHB, chronic hepatitis B; LC, liver cirrhosis; CC, cervical cancer; ddPCR, droplet digital PCR; NGS, next-generation sequencing; DFS, disease-free survival; OS, overall survival; VAF, variant allele frequencies; RFS, recurrence-free survival; HNSCC, head and neck squamous cell; PFS, progression-free survival; TFx, tumor fraction; TACE, transcatheter arterial chemoembolization; TKI, Tyrosine kinase inhibitors.

**Table 2:** Approaches for vh-DNA detection in cfDNA.

Approaches	Coverage	Sensitivity	Price	Limitation	Applications
WGS	Unbiased	+	++++	Extremely high output is required due to the low amount of tumor-specific vh-DNA in total cfDNA	Primary tumor detection Treatment response monitoring MRD recurrence detection Determine the clonality of recurrence or metastasis Identify putative carcinogenic mechanisms
Capture-NGS	Probe-enriched vh-DNA	++	+++	Detection results greatly depend on probe design and hybridization stringency	
ddPCR	Sequence-specific vh-DNA	++++	++	Sequence of target vh-DNA is required for the establishment of the assay Repetitive sequence in human genome might increase the difficulties in designing specific primer sets	Treatment response monitoring MRD recurrence detection
General PCR		+++	+		

**Table 3:** Experimental models of ctDNA in virus-related cancers.

Tumor	Aim	Experiment	Model	Animal	Cell Line	Target	Detection	Results	Ref.
HCC	Study the dynamics of cDNA release	Xenograft	Intratumor heterogeneity	Nude mice	Huh7, HepG2	hLINE	PCR	Tumor formed by different cell lines unevenly release ctDNA into the circulation.	[106]
						APOB mutation	ddPCR		
						FGA mutation	ddPCR		
HNSCC	Study the dynamics of cDNA release	Cell culture	Apoptosis, irradiation treatment	-	HMS-001, Vu147T, SCC090, FaDu, Cal33, PE/CA-PJ41, Cal27, BHY, SNU1076	hLINE	qPCR	Necrosis and apoptosis are the mechanisms contributing to the IR-induced release of ctDNA, while IR-induced cellular senescence counteract the release of ctDNA.	[107]
		Xenograft	Irradiation treatment	Nod-Scid-Gamma mice, Nod Rag Gamma mice	HMS-001, Cal33, Vu147T				
HNSCC	Study the dynamics of cDNA release	Xenograft	Surgical removal	New Zealand white rabbit	VX2	CRPV E6	qPCR	The level of ctDNA reflects the tumor burden.	[108]
HNSCC	Study the dynamics of cDNA release	Xenograft	Irradiation treatment	New Zealand white rabbit	VX2	CRPV E6	qPCR	The level of ctDNA reflects the tumor burden after IR treatment.	[109]

Abbreviations: CRPV, kappapapillomavirus 2.

## Data Availability

No new data were created or analyzed in this study. Data sharing is not applicable to this article.
